# Radiation enhanced the local and distant anti-tumor efficacy in dual immune checkpoint blockade therapy in osteosarcoma

**DOI:** 10.1371/journal.pone.0189697

**Published:** 2017-12-18

**Authors:** Yutaka Takahashi, Tomohiro Yasui, Keisuke Tamari, Kazumasa Minami, Keisuke Otani, Fumiaki Isohashi, Yuji Seo, Ryosuke Kambe, Masahiko Koizumi, Kazuhiko Ogawa

**Affiliations:** 1 Dept. of Radiation Oncology, Osaka University Graduate School of Medicine, Suita, Osaka, Japan; 2 Dept. of Medical Physics and Engineering, Osaka University Graduate School of Medicine, Suita, Osaka, Japan; ENEA Centro Ricerche Casaccia, ITALY

## Abstract

Radiation therapy has been long utilized as localized cancer treatment. Recent studies have also demonstrated that it has a distant effect by the enhanced immunity, but it rarely occurs. The purpose of this study was to investigate whether X-ray irradiation combined with anti-PD-L1 and anti-CTLA-4 antibodies (P1C4) provides a higher probability of this distant effect as well as enhanced local antitumor efficacy for osteosarcoma. LM8 mouse osteosarcoma cells were inoculated into both legs of C3H mice assigned to one of four groups, namely no treatment (No Tx), P1C4, X-ray irradiation (RAD) to the leg of one side, and combination (COMB) groups. Survival and treatment-related immune molecular changes were analyzed. Administration of P1C4 produced a tumor growth delay on day 30 in 18% of the mice. In contrast, combination therapy produced the strongest tumor growth inhibition not only at the irradiated tumor but also at unirradiated tumor in 67% of the mice. Accordingly, lung metastasis in the COMB group was strongly reduced by 98%, with a significant survival benefit. Unirradiated tumor in mice in the COMB group significantly recruited CD8 + tumor-infiltrating lymphocytes with a moderate reduction of Treg, producing a significant increase in the CD8/Treg ratio. These results suggest that radiation enhances the efficacy of P1C4 treatment against distant metastasis as well as local control in osteosarcoma. Our data suggest that radiation therapy combined with dual checkpoint blockade may be a promising therapeutic option for osteosarcoma.

## Introduction

Osteosarcoma is a common malignancy of bone in children and adolescents [1, 2]. Although multiagent chemotherapy and improved surgical techniques have increased overall survival to 65–70% [2–3], metastasis remains a barrier to further improvements in clinical outcome. To overcome this problem, a new therapeutic strategy for the control of metastasis is necessary.

Recently, immune checkpoint blockade has attracted attention as antitumor immune therapy for some kinds of tumor, including melanoma, non-small cell lung cancer, and breast cancer etc. in preclinical models and clinical trials [4–11]. The PD-L1/PD-1 pathway is known to be an inhibitor of T cell function. Specifically, increased expression of PD-L1 results in the loss of T cell effector function and a decrease in T cell proliferation [12–16]. Thus, the use of PD-1 or PD-L1 monoclonal antibodies strongly enhances antitumor immunity by inhibiting the exhaustion of cytotoxic T cells. On the other hand, the CTLA-4/B7-1 or CTLA-4/B7-2 pathway also plays an important role in the function of regulatory T cells (Treg), which are essential for maintaining homeostasis but which negatively regulate antitumor immunity [17].

Nevertheless, treatment of osteosarcoma still requires a strong local treatment strategy for primary tumors, including surgery or high precision radiation therapy to eradicate the cancer cells. Although osteosarcoma is known to be X-ray-resistant [18], a recent technical innovation in a high precision radiation therapy machine and treatment planning system allows for highly conformal beam delivery by intensity modulated and image-guided radiation therapy, providing a high radiation dose to the tumor while minimizing the dose to surrounding normal tissues. Radiation is therefore particularly useful for pediatric patients with osteosarcoma. In addition to its contribution to treatment of the primary tumor, regression of metastatic tumors outside the radiation field is also observed [19–22]. This phenomenon is called the abscopal effect. Although the abscopal effect has been seen in both preclinical models and clinical practice, it is nevertheless rare. Recently, however, several groups demonstrated that the combination of X-ray irradiation with immune checkpoint blockade provides a higher probability of the abscopal effect for some kinds of tumor [23–27]. However, the effect of X-ray irradiation combined with immune checkpoint blockade on the abscopal effect for osteosarcoma is totally unknown.

Here, we evaluated the efficacy of anti-PD-L1 and anti-CTLA-4 antibodies with X-ray irradiation in both local and distant effects against osteosarcoma.

## Materials and methods

### Ethics statement

Mice were maintained in a specific pathogen-free area in Osaka University. All experimental procedures were approved by the Osaka University Institutional Animal Care and Use Committee in accordance with the principals and procedures outlined in the Japanese Act on the Welfare and Management of Animals and the Guidelines for the Proper Conduct of Animal Experiments issued by the Scientific Council of Japan. All efforts were made to minimize animal suffering. For X-ray irradiation, mice were immobilized with 30 mg/kg of pentobarbital by intraperitoneal injection to fix mice on an in-house jig. Except for the irradiation, no more than mild or moderate discomfort of animals was expected from the treatments, and no unexpected discomfort was observed.

Mice were observed on a daily basis and humanely sacrificed by CO_2_ inhalation when they met the following humane endpoint criteria: prostration, skin lesions, difficulty in breathing, epistaxis, rotational motion, or the longest dimension of the tumor reached ≥15 mm.

### Cell lines

LM8 mouse osteosarcoma cell line was purchased from RIKEN (Saitama, Japan). This cell line underwent a microbiological examination in June 2015 by the Central Institute for Experimental Animals Monitoring Center in Kanagawa, Japan, and was found to be free of contamination by Mycoplasma using a PCR test. The cells were maintained in DMEM supplemented with 10% FBS, 5 mM penicillin/streptomycin, and L-glutamine at 37°C in a 5% CO_2_ atmosphere in an incubator.

### Mouse experiments

Seven-to-eight-week old C3H/HeNJcl mice were purchased from Nihon-Clea (Tokyo, Japan) and maintained in a specific pathogen-free area in Osaka University.

**Fig 1:**
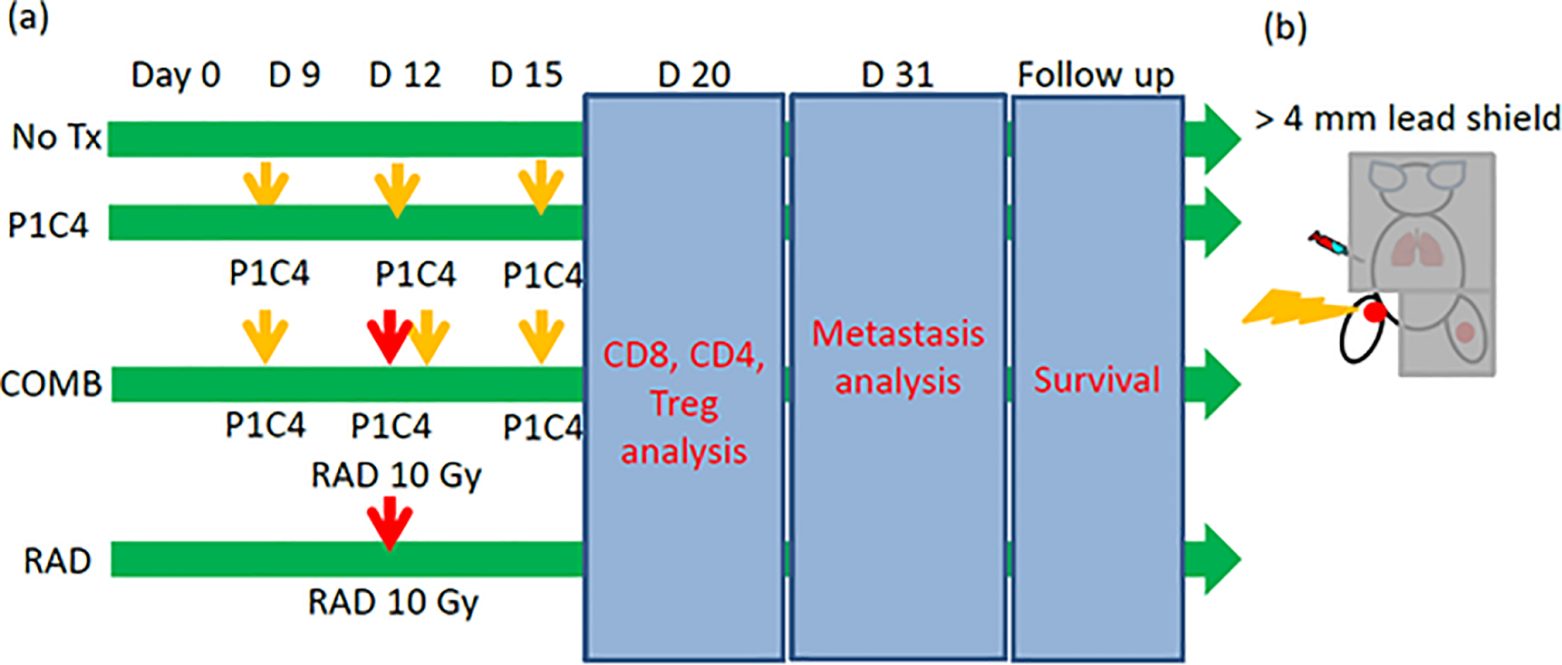
The experimental scheme. (a) Treatment schedule and endpoints. LM8 cells were inoculated into both legs on day 0. P1C4 were intraperitonealy injected on days 9, 12 and 15. Irradiation was performed on day 12. (b) Irradiation setup. One leg was irradiated while the remaining body was shielded with ≥4 mm lead blocks. Abbreviations:No Tx: No treatment; P1C4: Anti-PD-L1 and anti-CTLA-4 antibodies; COMB: Anti-PD-L1 and anti-CTLA-4 antibodies with X-ray irradiation; and RAD: X-ray irradiation.

The overall treatment and experimental scheme is shown in Fig 1(A). Sixty microliters of 3 x 10^5^ LM8 cells in PBS were inoculated into both legs of C3H mice which had been assigned to one of four groups: an untreated group (No Tx group); a group receiving anti-PD-L1 and anti-CTLA-4 antibodies on days 9, 12, and 15 (P1C4 group); a group receiving X-ray irradiation to a tumor on the leg of one side on day 12 (RAD group); and a combination group receiving both antibodies and X-ray irradiation (COMB group).

For the P1C4 and COMB groups, anti-PD-L1 (Clone: 10F.9G2) and anti-CTLA-4 (Clone; 9H10) antibodies (both BioXcell, NP, USA) were given by intraperitoneal injection at 150 ug on days 9, 12 and 15 after tumor inoculation. For the RAD and COMB groups, mice were immobilized with 30 mg/kg of pentobarbital by intraperitoneal injection and fixed on an in-house jig. The leg of one side of the mouse was then irradiated at 10 Gy while the rest of the mouse was protected with ≥ 4 mm lead blocks on day 12 using an orthovoltage X-ray irradiator (Rigaku Denki, Tokyo, Japan) under conditions of 180 kVp, 15 mA with a 1 mm Al filter at the animal experiment institution of Osaka University, as shown in Fig 1(B). The thickness of the lead shields over the irradiated mice was determined by radiation dose measurements using GafChromic EBT3 films (Ashland, KY, USA) to reduce the dose to ≤5% of the prescribed dose.

Tumor volume in mice in the No Tx (N = 13), P1C4 (N = 11), RAD (N = 9), and COMB (N = 12) groups was measured every three days at least using the formula *L* x *W*^2^ x 0.52, where *L*, and *W* represent the longest and the perpendicular dimensions, respectively. In this study, we defined tumor growth delay or partial response as when the volume on day 21 or day 30 was less than 3-fold that on day 9 when the first treatment with anti-PD-L1 and anti-CTLA-4 antibodies were given.

Lung, liver, and kidney metastases in mice in the No Tx (N = 8), P1C4 (N = 6), RAD (N = 5), and COMB (N = 5) groups were assessed on day 31 after tumor inoculation. Organs were fixed in 10% formalin and metastatic nodules were counted with a stereomicroscope as described previously [28]. Hematoxylin and eosin staining was performed in paraffin sections from these organs and the number and size of micrometastases were evaluated.

For the mice survival study, mice were from the cohorts of the tumor volume study and metastasis assay assigned to the P1C4 (N = 6), COMB (N = 7), and RAD (N = 6) groups. An event was defined as death when the mice met any of the humane endpoint criteria, including prostration, skin lesions, difficulty breathing, epistaxis, rotational motion, or the longest dimension of the tumor reached ≥15 mm. The Kaplan-Myer method was used to calculate survival and p-values were calculated using log-rank test and adjusted by the method of Holm.

### Flow cytometry

Proportion of CD8+ cells in unirradiated tumors in the No Tx (N = 7), P1C4 (N = 11), COMB (N = 9), and RAD (N = 5) groups was analyzed on day 20 after inoculation. We also analyzed Tregs and CD8 to Treg ratio in tumors in some of these mice (No Tx; N = 5, P1C4; N = 5, COMB; N = 3, RAD; N = 4).The tumors were surgically removed and minced in HBSS supplemented with 1% BSA. To make single cell suspensions, the minced tumor was dissociated in HBSS supplemented with 0.5 mg/ml of collagenase IV (Sigma Aldrich, Tokyo, Japan), 200 ug/ml of DNase (Sigma Aldrich, Tokyo, Japan) and 1% BSA at 37°C on a shaker for 90 min as described previously [29]. Anti-mouse CD16/32 antibody (Biolegend, CA, USA) was added as the Fc block for 10 min at room temperature before reaction with CD8 or CD4 antibodies. Anti-mouse rat CD8-APC antibody (Clone: 53–6.7, eBioscience) and anti-mouse Granzyme-B-PE (Clone: NGZB, eBioscience) were used for CD8 and Granzyme-B (GzmB) analysis in TILs, respectively. For Treg analysis in TILs, anti-mouse rat CD4-APC (Clone: RM4-5, eBioscience), anti-mouse rat FoxP3-PE (Clone: FJK-16s, eBioscience) antibodies, and FoxP3/Transcription Factor Staining Buffer Set (eBioscience) were used. All procedures in sample preparations were done according to the manufacturer’s instructions.

For in vitro PD-L1 expression analysis, cells were irradiated with a Gammacell (Shimazu, Kyoto, Japan). To examine dose dependency, multiple doses (2, 6, or 10 Gy) were used. After 72 hours, cells were harvested and reacted with anti-mouse CD274-PE antibody (Clone: MIH5, BD Pharmingen, NJ, USA).

The stained cells were analyzed with a FACS Verse^TM^ (BD, NJ, USA). Gating to identify lymphocytes was performed using splenocytes as shown in the supporting S1(A) Fig. The percentage of CD8 TILs or Tregs (CD4+ and FoxP3+) [25, 30] were then calculated. The analysis was performed using FlowJo ver.10 (Tommy Digital Biology, Tokyo, Japan).

### Quantitative RT-PCR

We conducted a single X-ray irradiation of LM8 cells at 2 or 10 Gy to evaluate gene expression of PD-L1 in vitro with a Gamma cell. Total RNA was extracted from the irradiated or unirradiated cells using an RNeasy Mini Kit (QIAGEN, Hilden, Germany) 72 hours after irradiation according to the manufacturer's manual. cDNA was then synthesized using ReverTra Ace qPCRRT Master Mix with gDNA Remover (Toyobo, Osaka, Japan). The gene-specific primer sequences were provided as follows:

PD-L1Fw: 5’-GACCAGCTTTTGAAGGGAAATG-3’

PD-L1 Rv: 5’-CTGGTTGATTTTGCGGTATGG-3’

Reactions were run on a ViiATM7 Real-Time PCR System (Applied Biosystems, CA, USA)

### Statistics

Irradiated and unirradiated tumor volumes in the No Tx, P1C4, RAD and COMB groups, and the number of metastatic nodules between the No Tx, P1C4, RAD and the COMB groups were compared using Tukey’s honestly significant difference test. Fisher’s exact test was performed to compare partial and complete responses, and progressive disease between the P1C4 and COMB groups. CD8, and Treg expressions and CD8/Treg between these groups were analyzed using the two-tail Student t-test with Bonferroni-corrected p-values. GzmB expression between the No Tx, P1C4, and COMB groups were analyzed using Dunnett’s multiple comparison test. Kaplan-Myer survival analysis was done using the log rank test with the adjusted p-values by the method of Holm for the multiple comparisons.

## Results

### Radiation enhanced both local and outside-radiation-field antitumor efficacy in anti-PD-L1 and anti-CTLA-4 therapy

To investigate the antitumor immune response to combined therapy for primary and distant tumors, we evaluated volume changes of irradiated and outside-radiation-field (unirradiated) tumors in the No Tx (N = 13), P1C4 (N = 11), RAD (N = 9), and COMB (N = 12) groups by day (V_d_) normalized to volume on day 9, the day of the first treatment (V_int_).

**Fig 2:**
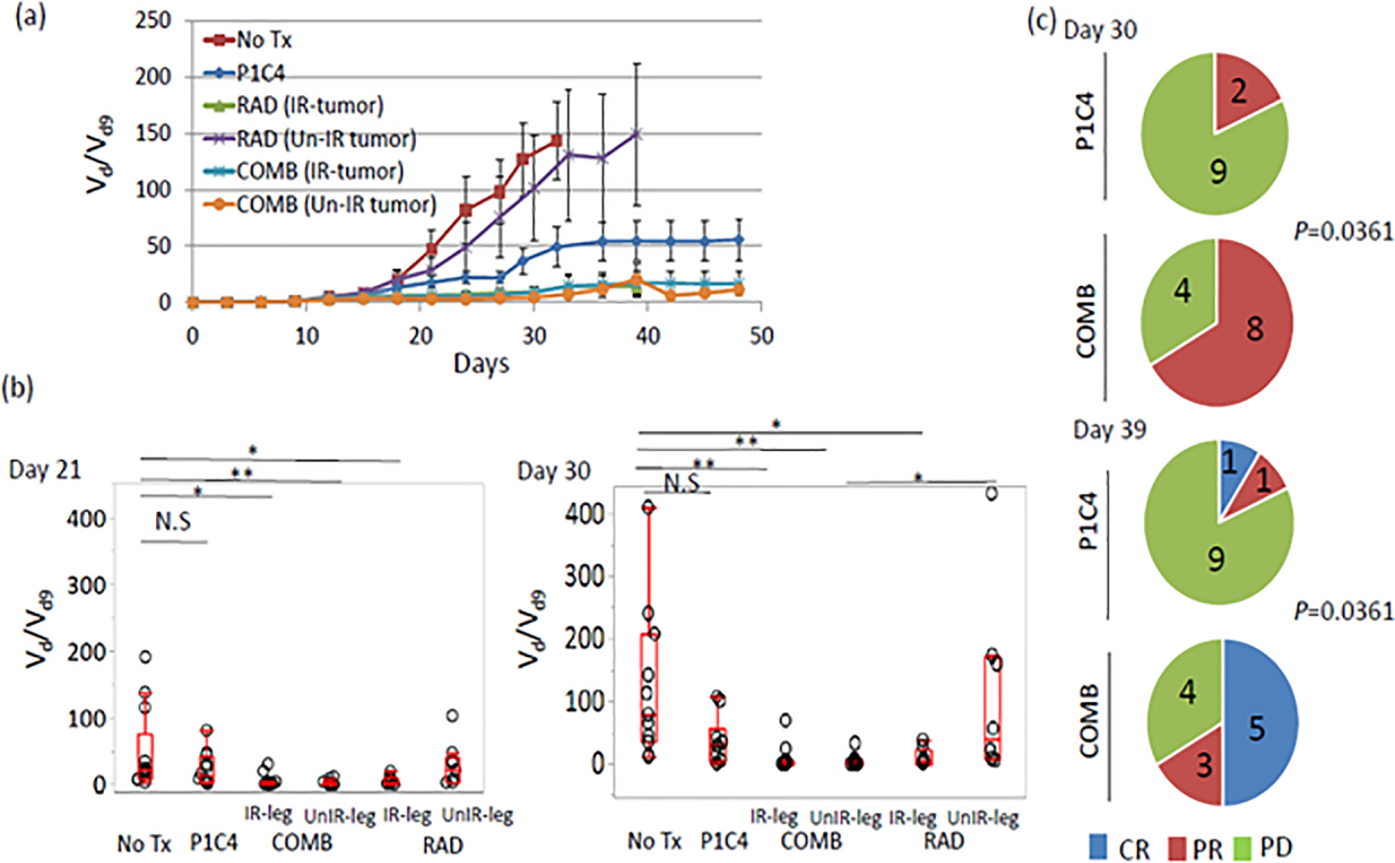
Effect of P1C4 and/or X-ray irradiation on tumor volume change in irradiated and unirradiated tumors. (a) Tumor volume change each day was normalized to volume on day 9 in the No Tx (N = 13), P1C4 (N = 11), X-ray irradiation (N = 9), and combination groups (N = 12). (b) Quantitative analysis of tumor volume change at day 21 and day 30. P-values were determined by Turkey’s honestly significant difference test. All data including the outlier were included in the statistical analysis. *, P<0.05. **, P<0.01. (c) Proportion of mice with partial response or complete response. The numbers in the pie chart indicate the number of mice. On day 30,2of 11 mice (18%) in the P1C4 alone group experienced a partial response whereas 8 of 12 mice (67%) in the combined therapy group had a partial response in the unirradiated tumor. On day 39, only 1 of 11 mice (9%) in the P1C4 group experienced complete response whereas 5 of 12 mice (42%) did so in the COMB group. *P*-values were determined by the Fisher’s exact test for comparison of the partial and complete response, and progressive disease between the P1C4 and COMB groups. Abbreviations: No Tx: No treatment; P1C4: Anti-PD-L1 and anti-CTLA-4 antibodies; COMB: Anti-PD-L1 and anti-CTLA-4 antibodies with X-ray irradiation; RAD: X-ray irradiation; IR-leg: Irradiated leg; UnIR-leg: Unirradiated leg, Vd: Volume on each day, Vint: Volume on the day of the initial treatment, CR: Complete response, PR: Partial response, and PD: Progressive disease.

Although mice in the No Tx group showed rapid tumor growth in tumors in both legs, intermediate tumor growth delay was observed in the P1C4 group (Fig 2(A)). Interestingly, anti-tumor efficacy was identical between irradiated and unirradiated tumors in mice of the COMB group and irradiated tumors in the RAD group. Quantitative analysis on days 21 and 30 revealed no significant differences in tumor volume change between the No Tx and P1C4 groups (Fig 2(B)). In contrast, mice in the COMB group experienced significant tumor volume reduction both in the irradiated and unirradiated tumors from day 21 (Fig 2(B)). Interestingly, further tumor volume reduction was observed even on day 30. In the RAD group, while irradiated tumor volume was significantly reduced, no abscopal effect was observed (Fig 2(A) and 2(B)).

To examine whether radiation increases the probability of the abscopal effect, we analyzed the number of mice which experienced partial response (PR), defined as when the volume on day 21 or day 30 was less than 3-fold that on day 9, or complete response (CR) in the unirradiated tumor. Two of 11 mice (18%) in the P1C4 group experienced PR on day 30 (Fig 2(C)). In contrast, a significantly higher PR rate (8 of 12 mice) was observed in the unirradiated tumor in mice in the COMB group compared with those in the P1C4 group (*P* = 0.0361). Surprisingly, this enhanced anti-tumor efficacy in the unirradiated tumors was extended by day 39. Specifically, 5 of 12 mice (42%) in the COMB group experienced CR but only 1/11 mice (9%) in the P1C4 group. These results suggest that the combination therapy induced abscopal effect in higher probability than the treatment of P1C4 alone.

### Radiation combined with anti-PD-L1 and anti-CTLA-4 therapy provided best suppression of distant metastasis and provided a survival benefit

**Fig 3:**
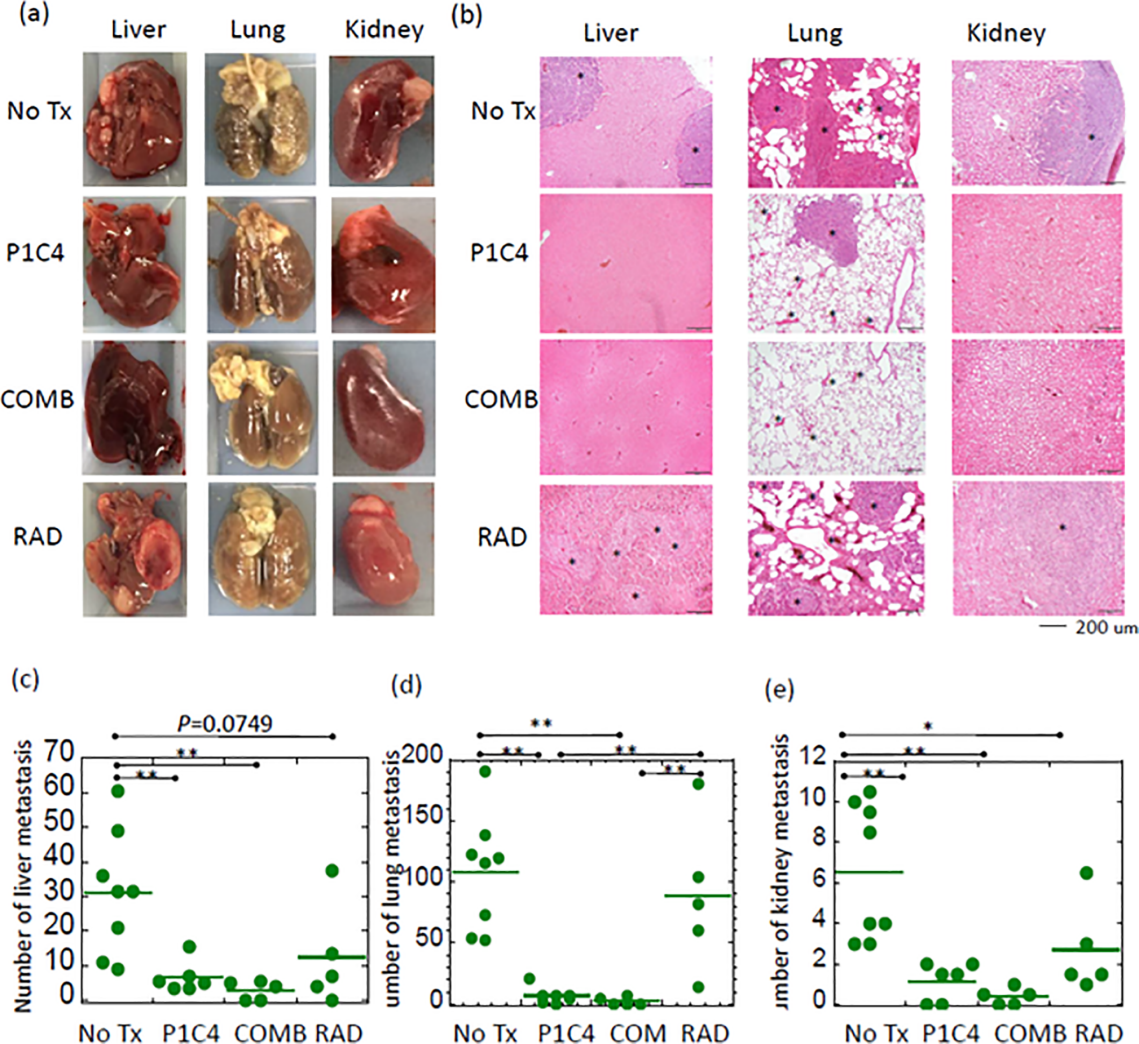
Effect of P1C4 and/or X-ray irradiation on distant metastasis. (a) Representatives of liver, lung and kidney metastases in the No Tx, P1C4, X-ray irradiation, and combination groups, (b) Hematoxylin-eosin staining for the corresponding organs in (a) (magnification; x100). Asterisk marks show representative micrometastases. Quantitative analysis of the number of gross metastatic nodules in the No Tx (N = 8), P1C4 (N = 6), X-ray irradiation (N = 5), and combination groups (N = 5) is shown for the (c) liver, (d) lungs, and (e) kidneys. Bars show the mean value. P-values were determined by Tukey’s honestly significant difference tests; *, P<0.05, **, P<0.01. Abbreviations: No Tx: No treatment; P1C4: Anti-PD-L1 and ani-CTLA-4 antibodies; COMB: Anti-PD-L1 and anti-CTLA-4 antibodies with X-ray irradiation; and RAD: X-ray irradiation.

To examine whether dual immune checkpoint blockade or combination therapy is effective in inhibiting metastasis, we assessed the number of metastatic nodules in lungs, kidneys and liver in mice in the No Tx (N = 8), P1C4 (N = 6), RAD (N = 5), and COMB (N = 5) groups on day 31 after tumor inoculation. As shown in Fig 3(A), fewer gross metastatic nodules were observed in the P1C4 and COMB groups in the liver, lung and kidneys. This trend was also confirmed in micrometastases (Fig 3(B)). Quantitative analysis of the number of gross metastatic nodules revealed that metastasis in the P1C4 and COMB groups was significantly reduced compared with mice in the No Tx group (Fig 3(C), 3(D) and 3(E)). Notably, lung metastasis in the P1C4 and COMB groups was strongly reduced by 94% (p = 0.0002) and 98% (p = 0.0005), respectively. X-ray irradiation inhibited lung and kidney metastases but did not significantly reduce metastasis to the liver compared with the No Tx group. Although differences in metastatic nodules in the liver, kidney, and lung between the P1C4 and COMB groups were not statistically significant, combined therapy reduced these nodules by more than half.

**Fig 4:**
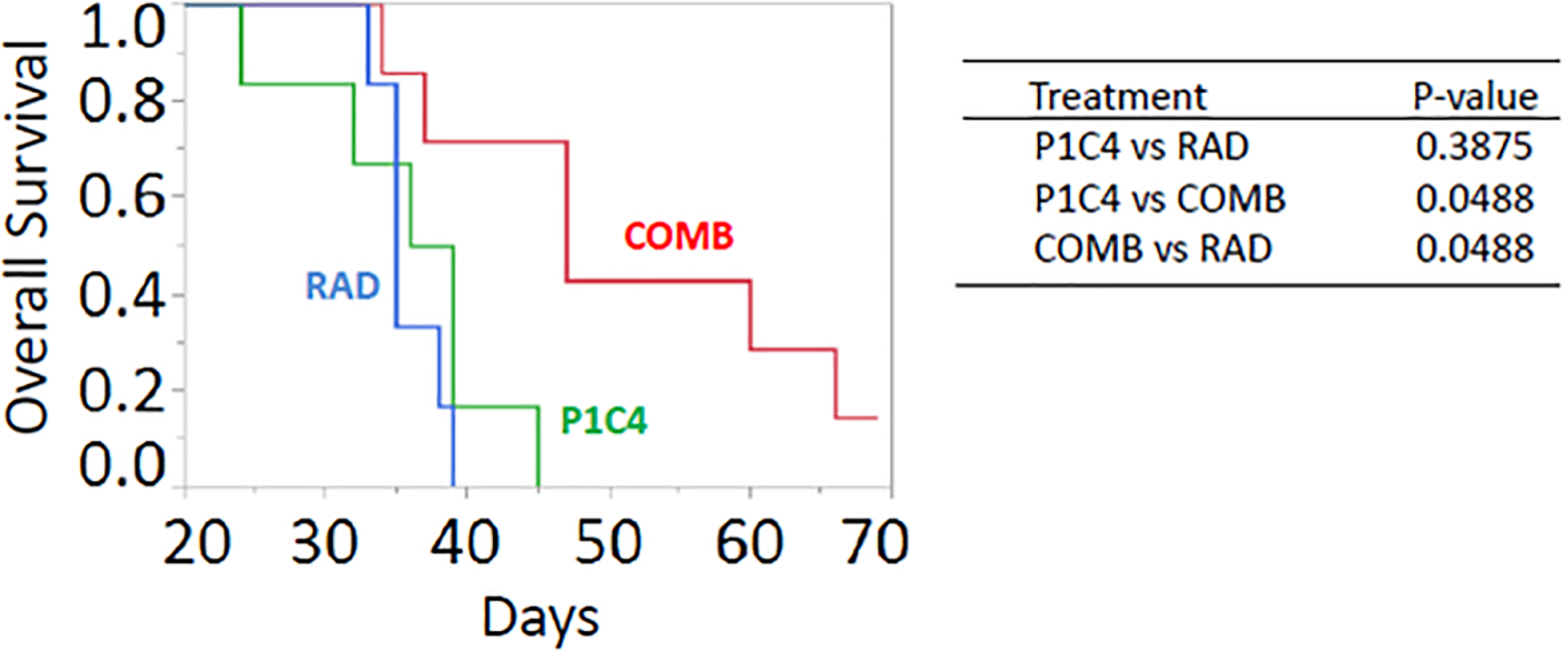
Effect of P1C4 and/or X-ray irradiation on overall survival by groups of the P1C4 (N = 6), RAD (N = 6), and COMB (N = 7). P-values were determined by the log-rank test with the adjustment by Holm method. Abbreviations: P1C4: Anti-PD-L1 and anti-CTLA-4 antibodies; COMB: Anti-PD-L1 and anti-CTLA-4 antibodies with X-ray irradiation; and RAD: X-ray irradiation.

We further investigated the impact of each treatment on long term survival. Despite the reduced distant metastases in the P1C4 therapy, combined therapy provided a significant benefit survival compared with the P1C4 and RAD groups (Fig 4). Mean survival was 35, 36, and 48 days for mice in the P1C4, RAD, and COMB groups, respectively. Although the longest survival in P1C4 mice was 45 days, 3 of 7 mice in the COMB group survived for ≥60 days. Moreover, one of the mice remained alive even on day 69, when follow up was ended.

### Radiation combined with anti-PD-L1 and anti-CTLA-4 therapy altered the infiltration of immune cells

As combination therapy provided a higher probability of the abscopal effect, we next examined immune-related molecular changes in the unirradiated tumors in mice in the No Tx, P1C4, COMB, and RAD groups.

**Fig 5:**
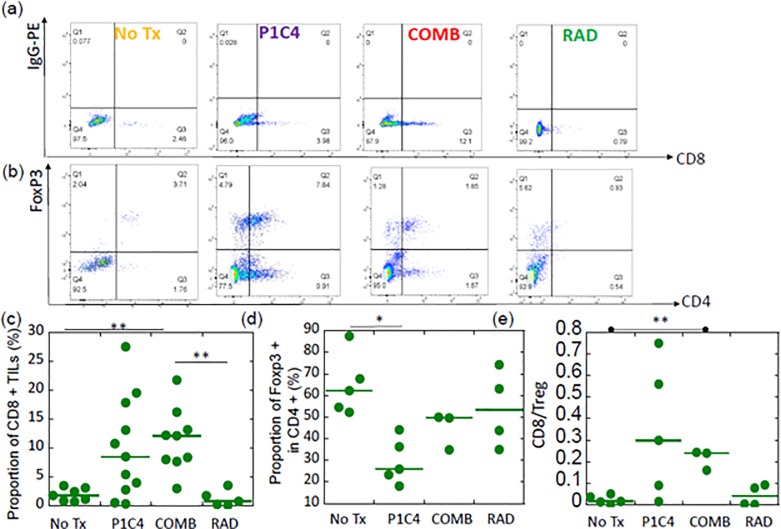
Changes in the tumor-infiltrated lymphocytes after treatment. (a) Representative plots of one animal per group for CD8 expression 20 days after tumor inoculation. (b) Representative plots of one animal per group for Treg expression 20 days after tumor inoculation. (c) Quantitative data of the proportion of CD8+ TILs. (d) Quantitative data of the proportion of FoxP3 + cells in CD4 + TILs. (e) Quantitative data of CD8 to Treg ratio. For CD8 analysis, the number of mice was 7, 11, 9, and 5 in the No Tx, P1C4, COMB, and RAD groups, respectively. Some of these mice were analyzed for Treg expression and CD8 to Treg ratio (No Tx; N = 5, P1C4; N = 5, COMB; N = 3, RAD; N = 4). P-values were determined by the Student t-test with Bonferroni correction; *, P<0.05, **, P<0.01. Bars show the median value. Abbreviations: No Tx: No treatment; P1C4: Anti-PD-L1 and anti-CTLA-4 antibodies; COMB: Anti-PD-L1 and anti-CTLA-4 antibodies with X-ray irradiation; and RAD: X-ray irradiation.

Fig 5(A) and 5(B) show the representative plots of a mouse per group for expressions of CD8+ cells and CD4+/ FoxP3+ cells in the unirradiated tumors in these groups, respectively. The corresponding data of the isotype control are provided in the S1(B) and S1(C) Fig. Flow cytometric analysis revealed large variation in CD8+ TILs in the P1C4-treated mice (Fig 5(A) and 5(C)), resulting in a non-significant increase in CD8+ TILs. Specifically, the proportion of CD8+ TILs in the P1C4 group was an average of 8.5%±7.7% (range; 0.5%-19.5%) in the unirradiated tumor. In contrast, as shown in Fig 5(C), the corresponding proportion in the COMB group was 9.5%±2.3% (range; 8.0%-12.1%), which was a significant increase compared to the No Tx group (p = 0.0118). Consistent with our finding that the abscopal effect was not seen in the RAD group, only a slight increase in CD8+ TILs was observed in unirradiated tumors in mice in the RAD group. We further examined proportion of GzmB, a cytotoxic protein within T cells, in unirradiated tumors in the No Tx (N = 8 tumors from 4 mice), P1C4 (N = 12 tumors from 6 mice), and COMB groups (N = 3 tumors from 3 mice). As shown in S2(A) and S2(B) Fig, significant increase of the CD8 +/GzmB + cells in the P1C4 groups was observed compared with the No Tx group (S2(B) Fig). Addition of radiation to P1C4 further increased the proportion of CD8 +/ GzmB + cells.

Tregs in the P1C4 group were significantly reduced (Fig 5(B) and 5(D)). Although the proportion of Treg in the No Tx group was an average of 64.9% ±14.1%, the corresponding proportion in the P1C4 group was 29.6%±10.5%, which was a significant decrease compared to the No Tx group (p = 0.0121). Slight decrease of Treg was observed in unirradiated leg in the COMB group. The CD8 to Treg ratio (CD8/Treg) in the P1C4 and COMB groups were 0.34 ±0.31 (range; 0.02–0.75) and 0.21±0.05 (range; 0.16–0.24), indicating that the range is wider in the P1C4 group than in the COMB group (Fig 5 (E)). Accordingly, the COMB group showed a significant increase in CD8/Treg compared to the No Tx group. These results suggest that a higher probability of the abscopal effect by radiation in P1C4 therapy may be associated with the altered infiltration of immune cells.

### Radiation induced PD-L1 on LM8 cells in vitro

**Fig 6:**
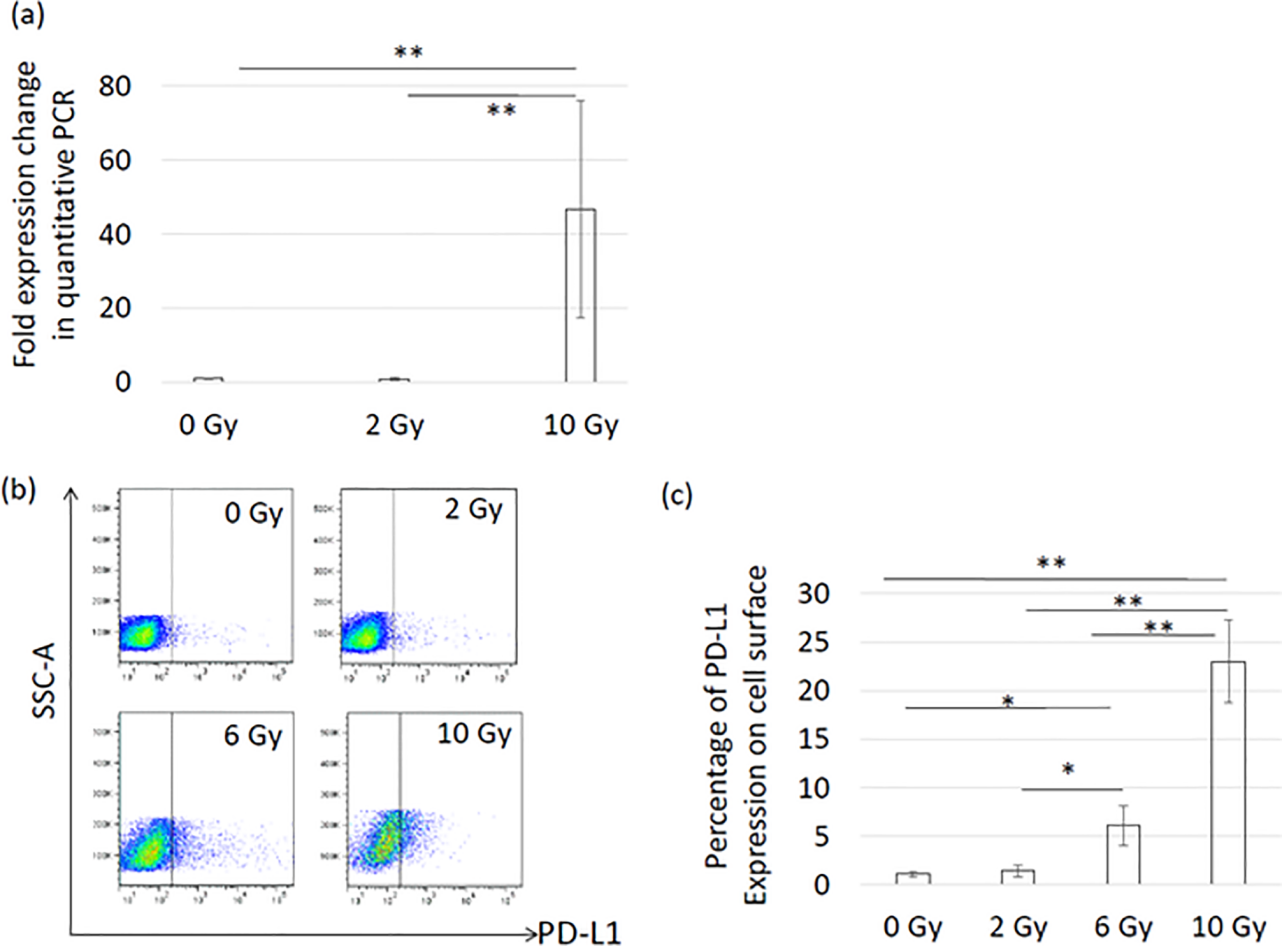
In vitro induction of PD-L1 72 hours after X-ray irradiation. (a) PD-L1 gene expression change analyzed by quantitative real-time PCR after X-ray irradiation. (b) Flow cytometric analysis of PD-L1 induction after X-ray irradiation. (c) Quantitative analysis of PD-L1 expression by flow cytometry after X-ray irradiation. All experiments were performed in triplicate or more. Error bars show standard deviation. P-values were determined by Tukey’s honestly significant difference tests; *, P<0.05, **, P<0.01.

To examine how X-ray irradiation might enhance the efficacy of anti-PD-L1 and anti-CTLA-4 antibodies, we investigated whether X-ray irradiation induced PD-L1 on the LM8 cells in vitro. As shown in Fig 6(A), quantitative real-time PCR showed that PD-L1 gene expression was strongly induced in a dose-dependent manner 72 hours after X-ray irradiation. In particular, a single fraction of 10 Gy increased PD-L1 by 47-fold. Consistent with this change in gene expression, these upregulations were also confirmed by flow cytometric analysis, as shown in Fig 6(B). In particular, high-dose irradiation significantly increased PD-L1 by more than 20-fold compared with untreated cells (Fig 6(C)).

## Discussion

The abscopal effect is rarely seen in radiation therapy and has even been considered anecdotal. However, induction of this phenomenon with high probability may lead to a great improvement in cancer therapy even for patients with distant metastasis. Recently, Dewan et al. demonstrated that anti-CTLA-4 antibody alone did not inhibit tumor progression or extend survival in metastatic osteosarcoma models compared to the no treatment mice (23). However, the addition of anti-PD-L1 antibody to CTLA-4- improved the response to metastatic tumors [23]. Victor et al. demonstrated that only 17% of B16-F10 melanoma mice responded to the CTLA-4 antibody with radiation [25]. We therefore hypothesized that the combination of anti-PD-L1 and anti-CTLA-4 antibodies, having non-redundant mechanisms for antitumor immune systems [25], with X-ray irradiation would provide a higher probability of the abscopal effect for osteosarcoma, which is known as an X-ray-resistant neoplasm.

Clinical trials for sarcoma are sparse and the effect of immune checkpoint blockade remains controversial. Maki et al. reported that ipilimumab, an anti-CTLA4 antibody, did not provide a survival benefit for synovial sarcoma patients [31]. To our knowledge, although a recent preclinical study using anti-PD-L1 and anti-CTLA-4 antibodies demonstrated good control of metastatic osteosarcoma [32], our present data provide the first direct evidence that the combination effect of anti-PD-L1 and anti-CTLA-4 antibodies with X-ray is the best therapeutic strategy for both the primary tumor and distant tumors or metastases in osteosarcoma.

First, we demonstrated that X-ray irradiation combined with anti-PD-L1 and anti-CTLA-4 antibodies provided faster and more prolonged regression in both irradiated and unirradiated tumors than that which occurred in the P1C4 group (Fig 2(A) and 2(B)). Furthermore, adding X-ray irradiation to P1C4 therapy significantly increased the probability of the abscopal effect (Fig 2(C)). Moreover, combined therapy strongly inhibited distant metastasis, producing the highest survival benefit. Second, these responses were associated with the higher recruitment of CD8 TILs by irradiation, lower Treg and higher CD8/Treg. Lastly, X-ray irradiation strongly induced PD-L1 expression in LM8 cells, which may be associated with increased antitumor efficacy.

Recently, Lussier et al. demonstrated that anti-PD-L1 antibody blockade provided longer survival with decreased pulmonary metastasis in a mice model of osteosarcoma [32]. Nevertheless, these mice eventually succumbed to pulmonary metastasis due to resistance to anti-PD-L1, in which other immune checkpoints, including anti-CTLA-4 antibody, may have been induced [33]. More recently, Lussier et al. demonstrated that the combination of anti-PD-L1 and anti-CTLA-4 antibodies provided complete control of metastasis in subcutaneously inoculated K7M2 osteosarcoma mice, despite the lack of any such effect when only anti-CTLA-4 antibody was administered [32]. Although this combination would be a great new treatment strategy, local antitumor efficacy for the primary tumor was not investigated [32]. In contrast, we found that mice in the P1C4 group showed no significant reduction in primary tumor volume, indicating the need for some kind of local therapy, including surgery or high precision radiation therapy. In the present study, we used a single high dose of non-invasive X-ray irradiation to treat the primary tumor. Interestingly, X-ray irradiation combined with P1C4 therapy contributed to the delay in tumor growth and even completely shrank the tumor, not only the irradiated tumor but also at the unirradiated tumor with higher probability than P1C4 only therapy. Consistent with the findings of Lussier et al. [32], our results also revealed the benefit for inhibition of distant metastases both in the P1C4 only and COMB groups on day 31. However, combination therapy significantly prolonged overall survival compared with the P1C4 groups. Because main cause of death in all mice (except for a mouse alive on day 69 in the COMB group) in the survival analysis was distant metastasis or large tumor burden in the primary sites, these results suggest that adding X-ray irradiation to the P1C4 therapy may extend the local and distant efficacy longer than P1C4 therapy alone. Our data provide the first evidence for a new treatment strategy for osteosarcoma. Advances in high-precision radiation therapy techniques now allow focused radiation delivery to the primary tumor with minimum irradiation of normal tissues. Radiation is therefore particularly useful in pediatric patients.

There are arguments on the timing of administration of immune checkpoint and radiation. Belcaid et al. [34] demonstrated that the treatment efficacy of radiation therapy with anti-CTLA-4 antibody is independent of the sequence in a murine glioma model. Victor et al. demonstrated that radiation given before and concurrently with CTLA-4 yields similar results in murine melanoma model [25]. They started antibody treatment on days 9, 12 and 15, and irradiation on day 12. In our osteosarcoma mice model, the tumor developed to a distinct size (>1mm) on day 9. We therefore chose the same time point as Victor et al [25]. However, the optimal timing of radiation and immune checkpoint blockade remains to be fully elucidated for other tumors, and further study is necessary.

Previous studies have explored various mechanisms of the abscopal effect, in which radiation enhances lymphocyte trafficking into the tumor microenvironment, induces tumor recognition and antigen presenting via danger signals in killing cells, increases cytokine secretion, and induces positive immunomodulatory pathways [19, 35–38]. Although localized radiation changes the tumor microenvironment and thus induces immune priming [39], Dovedi et al. demonstrated that the effect of radiation on tumor infiltrating lymphocytes is limited to irradiated sites [40]. Interestingly, addition of anti-PD-1 antibody to radiation significantly induced tumor-infiltrating CD8+ effector cells not only in irradiated tumors but also in out-of-field tumors, leading to the regression of both irradiated and out-of-field tumors [40], although the mechanism of how tumor-infiltrating lymphocytes were recruited to the out-of-field tumor remains unclear. Victor et al. demonstrated that radiation provided a modest increase in CD8 T cells in unirradiated tumor [25] and that the decrease in Tregs and increase in CD8 in unirradiated tumor were predominantly induced by anti-CTLA4 and anti-PD-L1, respectively [25]. Similarly, we found a significant increase in CD8 T cells and CD8/Treg ratio as well as moderate inhibition of Treg, not only in the irradiated tumor but also in the unirradiated tumor when anti-PD-L1 and anti-CTLA-4 antibodies were combined with X-ray irradiation. Taken together, radiation is necessary in P1C4 therapy to eradicate both primary and metastatic osteosarcoma. Further study is necessary to elucidate the mechanism of the absocopal effect including how immune cell trafficking is altered.

An increasing number of clinical trials using anti-PD-1, anti-PD-L1, and/or anti-CTLA-4 antibodies are ongoing. In a clinical trial, the expression level of PD-L1 was found to be associated with the enhanced efficacy of anti-PD-1 antibody [41]. Other research by Butte et al. demonstrated that the immune suppression signal is mediated via PD-L1-CD80 interactions as well as PD-L1-PD-1 interactions, indicating that anti-PD-L1 would be a better immune checkpoint blockade treatment option than anti-PD-1 [42]. Moreover, our results clearly showed that x-ray irradiation strongly induced PD-L1 on LM8 cells in a dose-dependent manner, suggesting that high dose X-ray irradiation enhances the efficacy of anti-PD-L1 and anti-CTLA-4 antibodies, mediated by the upregulation of those molecules.

In conclusion, we found that high dose radiation was necessary in anti-PD-L1 and anti-CTLA-4 therapy for local control as well as inhibition of distant metastasis in this mouse model of osteosarcoma, and that this treatment leads to the longest overall survival. Our data suggest that radiation therapy combined with dual checkpoint blockade may be a promising therapeutic option for osteosarcoma. Further studies on the timing of the administration of immune checkpoint blockade, optimal radiation delivery regimen including total dose and fractionation, the mechanism of the abscopal effect including deep analysis of the immune cell function, and chemokine expressions will provide a strategy to increase the probability of the abscopal effect.

## Supporting information

S1 Fig Analysis of immune cells in unirradiated tumors. (a) Gating strategy to identify lymphocytes. Splenocytes were harvested and a Forward Scatter (FSC)-Side Scatter (SSC) plot was generated to identify the lymphocytes. The gate was adapted to the FSC-SCC plot of the tumors. (b) The dot plots of isotype control corresponding to Fig 5 (A). The gate of the P1C4 and COMB groups was shared because the experiment was conducted in the same day under the exactly same condition. (c) The dot plots of isotype control corresponding to Fig 5 (B).
Abbreviations:
No Tx: No treatment; P1C4: Anti-PD-L1 and ani-CTLA-4 antibodies; COMB: Anti-PD-L1 and anti-CTLA-4 antibodies with X-ray irradiation; and RAD: X-ray irradiation.
(TIF)

S2 Fig Proportion of CD8+/GzmB+ cells in unirradiated tumors in the No Tx (N = 8 tumors from 4 mice), P1C4 (N = 12 tumors from 6 mice), and COMB groups (N = 6 tumors from 6 mice). (a) Representative dot plots of one animal per group for proportion of CD8+/GzmB+ cells 20 days after tumor inoculation. (b) Quantitative data of proportion of CD8+/GzmB+ cells. P-values were determined by Dunnett’s multiple comparison test. *, P<0.05. Bars show the median value.
Abbreviations:
GzmB: Granzyme-B; No Tx: No treatment; P1C4: Anti-PD-L1 and ani-CTLA-4 antibodies; COMB: Anti-PD-L1 and anti-CTLA-4 antibodies with X-ray irradiation.
(TIF)
