# Supplementary figures and images for: Radiation enhanced the local and distant anti-tumor efficacy in dual immune checkpoint blockade therapy in osteosarcoma
Source: PLoS One. 2017 Dec 18;12(12):e0189697. doi: 10.1371/journal.pone.0189697 (PMC5734786; doi:10.1371/journal.pone.0189697)

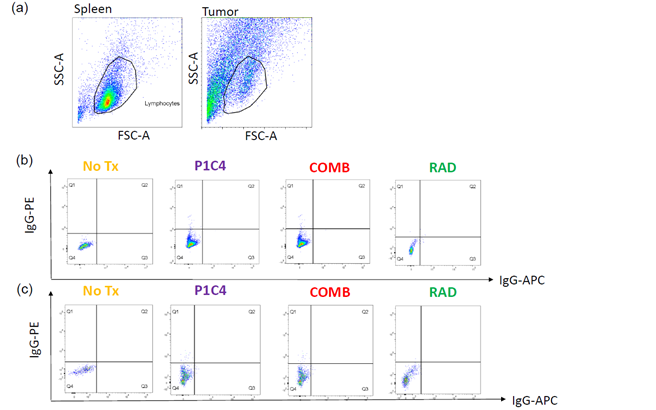

Supplement: S1 Fig — (a) Gating strategy to identify lymphocytes. Splenocytes were harvested and a Forward Scatter (FSC)-Side Scatter (SSC) plot was generated to identify the lymphocytes. The gate was adapted to the FSC-SCC plot of the tumors. (b) The dot plots of isotype control corresponding to Fig 5 (A). The gate of the P1C4 and COMB groups was shared because the experiment was conducted in the same day under the exactly same condition. (c) The dot plots of isotype control corresponding to Fig 5 (B). Abbreviations: No Tx: No treatment; P1C4: Anti-PD-L1 and ani-CTLA-4 antibodies; COMB: Anti-PD-L1 and anti-CTLA-4 antibodies with X-ray irradiation; and RAD: X-ray irradiation. (TIF) [file pone.0189697.s001.tif]

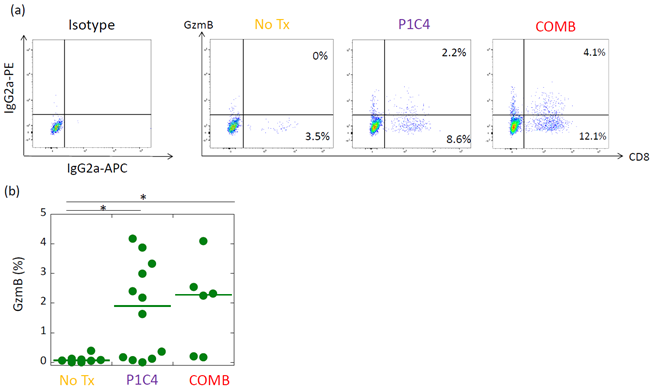

Supplement: S2 Fig — (a) Representative dot plots of one animal per group for proportion of CD8+/GzmB+ cells 20 days after tumor inoculation. (b) Quantitative data of proportion of CD8+/GzmB+ cells. P-values were determined by Dunnett’s multiple comparison test. *, P<0.05. Bars show the median value. Abbreviations: GzmB: Granzyme-B; No Tx: No treatment; P1C4: Anti-PD-L1 and ani-CTLA-4 antibodies; COMB: Anti-PD-L1 and anti-CTLA-4 antibodies with X-ray irradiation. (TIF) [file pone.0189697.s002.tif]
